# Complete Genome Sequence of the Solvent Producer *Clostridium saccharobutylicum* NCP262 (DSM 13864)

**DOI:** 10.1128/genomeA.00997-13

**Published:** 2013-11-27

**Authors:** Anja Poehlein, Katrin Hartwich, Preben Krabben, Armin Ehrenreich, Wolfgang Liebl, Peter Dürre, Gerhard Gottschalk, Rolf Daniel

**Affiliations:** Genomic and Applied Microbiology and Göttingen Genomics Laboratory, Georg-August University, Göttingen, Göttingen, Germanya; Green Biologics Ltd., Milton Park, Abingdon, Oxfordshire, United Kingdomb; Lehrstuhl für Mikrobiologie, Technische Universität München, Freising, Germanyc; Institut für Mikrobiologie und Biotechnologie, Universität Ulm, Ulm, Germanyd

## Abstract

*Clostridium saccharobutylicum* was employed for the production of acetone and butanol in South Africa until the 1970s. The genome comprises a single replicon (5,107,814 bp) harboring all the genes necessary for solvent production and the degradation of various organic compounds, such as fructose, cellobiose, sucrose, and mannose.

## GENOME ANNOUNCEMENT

The Gram-positive anaerobic spore-forming bacterium *Clostridium saccharobutylicum* is able to produce solvents, such as acetone, butanol, and ethanol (ABE), from various sugar compounds, such as fructose, cellobiose, sucrose, and mannose. It is the fourth clostridial species, after *Clostridium acetobutylicum*, *Clostridium beijerinckii*, and *Clostridium saccharoperbutylacetonicum*, to be described with this feature (1–3). *C. saccharobutylicum* NCP262 (DSM 13864) was used as a production strain by National Chemical Products in an ABE plant in South Africa until the late 1970s. It has been deposited as a type strain in several culture collections.

The MasterPure complete DNA purification kit (Epicenter, Madison, WI) was used to isolate the chromosomal DNA of *C. saccharobutylicum* NCP262 (DSM 13864). The genome sequence was determined by employing a 454 GS-FLX pyrosequencing system (Roche Life Sciences, Mannheim, Germany). Shotgun and paired-end libraries were prepared according to the protocols of the manufacturer (Roche). Sequencing resulted in 691,711 total reads containing 66,810 paired reads. Assembly was performed *de novo* with the Roche Newbler assembly software 2.0 and resulted in 6 scaffolds with 174 contigs. The average coverage is 29.07-fold. Gap closure was done by PCR-based approaches, Sanger sequencing of the PCR products, and use of the Gap4 (version 4.11) software of the Staden package (4). The complete genome of *C. saccharobutylicum* NCP262 (DSM 13864) comprises a single chromosome of 5.10 Mb, with an overall G+C content of 28.6%. The genome of *C. saccharobutylicum* is larger than that of *C. acetobutylicum* ATCC 824 (4.13 Mb) but smaller than those of *C. beijerinckii* NCIMB8052 (6.00 Mb) and *C. saccharoperbutylacetonicum* DSM 14923 (6.62 Mb). Automatic gene prediction was performed using the software tools YACOP and Glimmer (5). Identification of rRNA and tRNA genes was done with RNAmmer and tRNAscan, respectively (6, 7). The IMG-ER (integrated microbial genomes-expert review) system (8, 9) was used for automatic annotation, and the annotation was subsequently manually curated by using the Swiss-Prot, TrEMBL, and InterPro databases (10). We identified 12 rRNA operons, 85 tRNA genes, 3,160 protein-coding genes with function predictions, 1,233 genes coding for hypothetical proteins, and 37 pseudogenes. The *sol* operon consists of genes encoding aldehyde dehydrogenase (*ald*), coenzyme A (CoA) transferase (*ctfAB*), and acetoacetate decarboxylase (*adc*). The gene organization of the *C. saccharobutylicum sol* operon is identical to those of *C. beijerinckii* and *C. saccharoperbutylacetonicum* (11, 12) but differs from that of *C. acetobutylicum* ATCC 824, in which the aldehyde dehydrogenase gene is replaced by an alcohol-aldehyde dehydrogenase gene (*adhE*). In addition, the operon is located on a megaplasmid in *C. acetobutylicum* (13, 14). Genes coding for other key enzymes of ABE fermentation, such as acetyl-CoA acetyltransferase, crotonase, butyryl-CoA dehydrogenase, phosphate butyltransferase, butyrate kinase, phosphate acetyltransferase, acetate kinase, butyraldehyde dehydrogenase, and several alcohol dehydrogenases, are also present in the genome of *C. saccharobutylicum*. In addition, we identified several putative genes encoding phosphotransferase systems with different predicted substrate specificities, i.e., uptake systems for d-glucosamine, cellobiose, mannose, fructose, sucrose, lactose, β-glucosides, and l-ascorbate.

### Nucleotide sequence accession number.

The genome sequence has been deposited in GenBank under the accession no. CP006721.
